# Switching from offline to online health consultation in the post-pandemic era: the role of perceived pandemic risk

**DOI:** 10.3389/fpubh.2023.1121290

**Published:** 2023-05-16

**Authors:** Xue Pan, Xuecheng Zhou, Lei Yu, Lei Hou

**Affiliations:** School of Management Science and Engineering, Nanjing University of Information Science and Technology, Nanjing, China

**Keywords:** online health consultation, perceived pandemic risk, switching intention, switching behavior, push-pull-mooring framework

## Abstract

**Introduction:**

Due to its effectiveness and various benefits, the use of online health consultation (OHC) has dramatically increased in recent years, especially since the outbreak of the COVID-19 pandemic. However, underlying mechanism whereby the pandemic impacted OHC usage is still unclear.

**Methods:**

Via an online survey (N=318), the present paper measures the users’ perceptions towards both offline and online services, their intention to switch to OHC, and the perceived pandemic risks. The relationships among these factors are conceptualized by the push-pull-mooring framework, and tested via structural equation modelling.

**Results:**

Dissatisfaction with offline service (process inefficiency and consultation anxiety), the attractiveness of OHC (perceived benefits and perceived ease of use), and users’ behavioral inertia (switching cost and habit) jointly influence the intention to switching to OHC. The significant role of the perceived pandemic risk of going to medical facilities is particularly addressed. On the one hand, the perceived pandemic risk is found with an indirect impact on the switching intention by enlarging the dissatisfaction with offline service and the attractiveness of OHC. On the other hand, a high perceived pandemic risk induces more actual switching behavior and also amplifies the transition from switching intention to behavior.

**Discussion:**

The study provides novel insights into the understanding of OHC usage in the post-pandemic era, and also informs medical facilities, OHC platforms, and policymakers on managing and balancing the online and offline healthcare provision.

## 1. Introduction

Healthcare is an essential part of people’s well-being, but the traditional model of healthcare delivery suffers from inefficiency and inequality problems due to limited resources (1, 2). The advancing information technology enables the emergence of online health services as an extension to offline services (3). It is reported that online health services have significantly decentralized the distribution of healthcare resources by virtually moving the resources from big cities to underdeveloped regions, thereby easing inequalities (4). Due to the benefits of OHC, patients are increasingly choosing online health services for mild illnesses or chronic conditions (5, 6). For example, according to the 49th Statistical Report on Internet Development in China, online health services in China saw a growth of 38.7% in active users in 2021 reaching a population of 298 million users.

Online health consultation (OHC) is a typical online health service that enables patients to virtually consult physicians through online platforms and get diagnoses and prescriptions. OHC largely overcomes the barriers of distance and time and thus has been argued to be convenient, flexible, and efficient (7). As such, how patients perceive OHC and what factors drive patients to adopt OHC have become critical research questions that have caught heated attention. The perceived ease of use and perceived benefits of OHC are the most acknowledged factors that encourage adoption intentions (8–10). On the other hand, user-related factors are also investigated, such as user habits and social influence (11, 12). Attending a bricks-and-mortar hospital or medical clinic has been the default option for patients prior to the emergence of OHC. The adoption of OHC is potentially at expense of reducing visits to offline medical facilities and thus can be regarded as a switching behavior that an individual chooses to use OHC instead of their previous option of offline consultation. To make the switching decision, patients are likely to compare the two services. For example, patients who are dissatisfied with offline services are more likely to adopt OHC. As such, the adoption of OHC is potentially influenced by not only the OHC-related and user-related factors, but also the factors regarding offline services, such as the factors that may induce patient dissatisfaction. However, the offline service factors have received not enough attention. Although Zhang et al. (3) reported the negative effect of offline service satisfaction on OHC awareness, the factors still need to be further contextualized. The antecedents for switching from offline to online health consultation can be potentially framed by the push-pull-mooring theoretical framework, where the dissatisfactions with offline consultation push users away, the attractiveness of OHC pulls users to adopt the alternative, and the mooring factors such as costs and habits keep users maintaining their current behavior. However, how the previously identified influential factors could fit into the push-pull-mooring framework, and to what extent the framework could describe the adoption of OHC, have not been fully explored.

The outbreak of COVID-19 posed pressing challenges for a wide range of people’s daily activities, such as food choices (13) and education (14). Healthcare systems were particularly affected in various ways. To manage the risks brought by the pandemic, many healthcare systems were encouraged to deliver health services via online venues (15, 16). For example, the Chinese government issues a series of policies since the outbreak of COVID-19 to encourage the establishment of Internet hospitals, which offer online services including consultation, medical prescription, drug delivery, and so on (17). As a consequence, both the number of Internet hospitals and user digital visits vastly increased in the past years (5, 18). Although the increase in OHC adoption since the pandemic outbreak has been empirically observed and widely acknowledged, the underlying mechanism of the pandemic’s influence on people’s intention and behavior regarding OHC adoption is still unclear. With the mitigation of the pandemic, health services have largely returned to a pre-pandemic baseline of practice. In such a post-pandemic era, is the potential pandemic risk still a stimulus for switching from offline to online health consultation?

This study adopts the push-pull-mooring theoretical framework to explore the influential factors that drive people’s intention and behavior of switching from offline to online health consultation. The process inefficiency and consultation anxiety of offline consultation, the perceived benefits and perceived ease of use of OHC, and the switching cost and habit of users are regarded as push, pull, and mooring factors, respectively. As such, the factors of different stakeholders are integrated into a single framework. The impact of the perceived pandemic risk on the intention and behavior of switching is particularly explored, which offers novel insights into the understanding and practice of OHC.

## 2. Literature review

### 2.1. Online health consultation

The rapidly developing Internet technology has made a profound impact on the delivery of healthcare. Online health services, sometimes referred to as e-health, are health-related services delivered via online venues. Basically, the services of brick-and-mortar medical facilities, are transferred to an online option, becoming “internet hospitals” (18, 19). Various services are provided such as health information, appointments for offline services, consultation, diagnosis, and so on.

OHC is one of the most implemented and used online health services which enables patients to communicate their health conditions and symptoms to physicians via online systems (20). Especially with the support of online chatting and online video conferencing, OHC can largely reduce the need for face-to-face interviews, and thus is regarded as an effective, accessible, and cost-effective model of healthcare delivery (21). Physicians can thus telematically make diagnoses and prescriptions for the users according to the gathered information. The long-distance healthcare delivery can link doctors in developed regions to patients in underdeveloped regions. Such an effect is increasingly prominent with more and more physicians and patients joining OHC platforms over the past decades. As such, OHC has become an effective approach to redistributing healthcare resources, and easing regional disparities (4). Besides the improved accessibility, OHC is also argued to be convenient, flexible, and cost- and time-saving (7). Due to these various advantages, users, in general, are satisfied with OHC (20, 22). In Ping An Good Doctor, one of the biggest OHC platforms in China, 93% of the consultations are rated the highest scores (5 out of 5) by the users (5), indicating a high level of satisfaction among users. A survey among healthcare professionals also indicates that, although professionals are concerned about technological and usability problems, and difficulties in establishing rapport with clients, they do acknowledge the advantages of flexibility, improved patient contact, and reduced travel time (15).

OHC is not designed to fully replace, but to complement offline health services. Especially for low-risk or chronic diseases, patients can choose OHC to take advantage of its convenience and cost efficiency (5, 7). Normally, the OHC platforms are linked to offline health services through hospitals or physicians. Patients can always visit a medical facility or physician in person after consulting online. The intention of such offline visits is also largely determined by the OHC characteristics, such as the information quality (23) and the quality of doctor-patient online communication (24). In addition, OHC can be used as a follow-up service that a patient continues to consult his/her physician via online channels after offline diagnosis (25).

### 2.2. Adoption of OHC

With OHC offering healthcare system, patient and practitioner advantages, many governments have issued polices to encourage the development and use of OHC platforms. Consequently, many governments have issued policies to encourage the development and use of OHC platforms (17, 26). However, OHC platforms still suffer from a relatively low usage rate (12). Factors that drive users’ OHC adoption have thus been widely studied, which are summarized in Table 1.

**Table 1:** Summary of influential factors for the use of OHC in literature.

Study	Theoretical framework	Offline service factors	Online service factors	Other factors	Dependent variable
Jung and Padman (7)	None			Young age (−); Female (+).	Adoption of OHC
Yang et al. (27)	Service Quality Theory		Response timeliness (+); Interaction frequency (+).	Disease risk (*).	Satisfaction with OHC
Hoque and Sorwar (8)	Unified Theory of Acceptance and Use of Technology		Performance expectancy (+); Effort expectancy (+).	Social influence (+); Technology anxiety (−); Resistance to change (+).	Adoption of mobile health services
Zhang et al. (3)	Innovation Diffusion Theory	Satisfaction (−).	Awareness (+).	Healthcare habit (*).	Adoption of OHC
Zhang et al. (12)	Status Quo Bias Theory		Transition cost (−); Privacy protection beliefs (−).	Sunk cost (−); Healthcare habit (−).	Adoption of OHC
Gong et al. (11)	Extended Valence Framework		Perceived benefits (+); Trust (+).	Subjective norm (+); Healthcare habit (−).	Adoption of OHC
Zhao et al. (9)	Meta-analysis		Perceived usefulness (+); Perceived ease of use (+).	Subjective norm (+); Reginal economic development (*).	Adoption of online health services
Chang et al. (24)	Stimulus-Organism-Response Framework		Trust (+); Satisfaction (+).		Continued intention to OHC
Li et al. (25)	Elaboration Likelihood Model		Service quality (+); Patients votes (+); Private doctor service (*).	Disease privacy (*).	Adoption of online follow-up service
Zheng et al. (10)	Extended Valance Framework		Service quality (+); Perceived benefits (+); Trust (+); Perceived risks (−).		Adoption of OHC
This study	Push-Pull-Mooring Framework	Process inefficiency; Consultation anxiety.	Perceived benefits; Perceived ease of use.	Switching cost; Healthcare habit; Perceived pandemic risk.	Switching to OHC

− Negative effect; + positive effect; *moderation effect.

How users perceive OHC is found closely related to the adoption intention and behavior. On the one hand, if users perceive OHC to be useful or beneficial, they are more likely to adopt OHC (8–11). On the other hand, the perceived ease of use is also positively associated with OHC adoption (8, 9). This can also be conceptualized as transition cost, which is the opposite of ease of use. If OHC adoption is difficult and time consuming, users are less likely to do so (12). Service quality of OHC, such as technical quality (e.g., professional service) and interpersonal quality (e.g., response timeliness and interaction frequency), also significantly promotes satisfaction (27) and further encourages the adoption of OHC (10, 25) and continued use of OHC (24). Additionally, since OHC is a relatively new model of healthcare delivery, trust also plays a significant role in its adoption (10, 11, 24).

The social environment, and in particular social influence (8) and subjective norms (9, 11), appear to impact OHC adoption. Basically, if users’ peers have a positive attitude or experience with OHC, they are more likely to adopt OHC. For many people, going to a offline medical facility for health services has become a habit, which also has either a direct and negative impact on OHC adoption (11, 12) or a moderation effect on the other factors’ impact (3). The type of disease, such as low- versus high-risk diseases (27) and low- versus high-privacy diseases (25), is another factor that has been found to moderate the impact of other factors.

Although OHC cannot fully replace offline medical facilities, for some circumstances, such as minor illness which do not require sophisticated diagnosis, OHC can be regarded as an alternative to offline health services. When choosing between OHC and offline services, patients may consider not only the OHC-related factors but also the offline-related factors. For example, the habit of attending offline medical facilities has been confirmed to prevent users from adopting OHC (11, 12). However, previous literature has primarily focused on factors of OHC and users, while the factors related to offline medical facilities are less explored. Zhang et al. (3) measured the users’ satisfaction with offline healthcare according to service, system, and information satisfaction, which is reported to have a negative impact on the use intention of OHC mediated by the awareness of OHC. However, the direct impact of offline health services on OHC adoption is still unclear and an overarching framework integrating factors from offline health services, OHC, and users is still lacking.

### 2.3. Push-pull-mooring theoretical framework

Offline, or face-to-face services are the traditional mode of delivery of healthcare, while OHC emerged only in recent decades as an alternative. The OHC adoption can thus be regarded as a behavior of switching from offline to online health services. For explorations of such switching behavior, the Push-Pull-Mooring (PPM) model is among the most-used theoretical frameworks. The PPM model was originally proposed to investigate population migration from one place to another (28). As conceptualized by the model, dissatisfaction with the original place pushes people away, while the attractiveness of the alternative place pulls people to migrate. Meanwhile, various barriers to migration are believed to play a mooring effect that makes people keep their current status.

The PPM model has found its applications in the switching of consumers among different services. Generally, the push factors are expected to be negative characteristics of the original service, while the pull factors are the positive characteristics of the alternative service. However, the detailed push and pull factors could vary vastly across different contexts because these factors are expected to best describe the features of the studied services. For example, when studying the switching behavior from blogging to microblogging services, the long textual content for blogging is regarded as a push factor, while the short content in microblogging is regarded as a pull factor (29). Similarly, the fact that blogging services are suffering user loss also plays a push effect (30). In contrast, when examining the drivers for switching to anonymous social networking sites, the privacy concern is found to push users away and the anonymity of the alternative pulls users to switch (31). The perceived benefits and perceived ease of use for the alternative service are two widely considered pull factors, which describe users’ beliefs of the benefits and effort required to adopt the service (30, 32, 33). On the other hand, a mooring factor is expected to prevent users from switching, in other words, reflecting the inertia in the status quo. The mooring factors normally include the switching or sunk cost and the developed habit of using the original service (34, 35).

The model is also suitable for describing users’ switching behavior from offline to online services. For example, Chen and Keng (36) explored the switching behavior from offline to online English learning. The relative inconvenience, questionable quality, and higher price of offline learning act as push factors, while e-learning motivation and perceived quality of online learning act as pull factors as users contemplate switching. Meanwhile, learning engagement in the offline setting, social interaction available in offline settings, and switching costs act as mooring factors weakening both push and pull effects as well as having a direct impact on users’ switching intentions.

## 3. Research framework and hypotheses development

**Figure 1:**
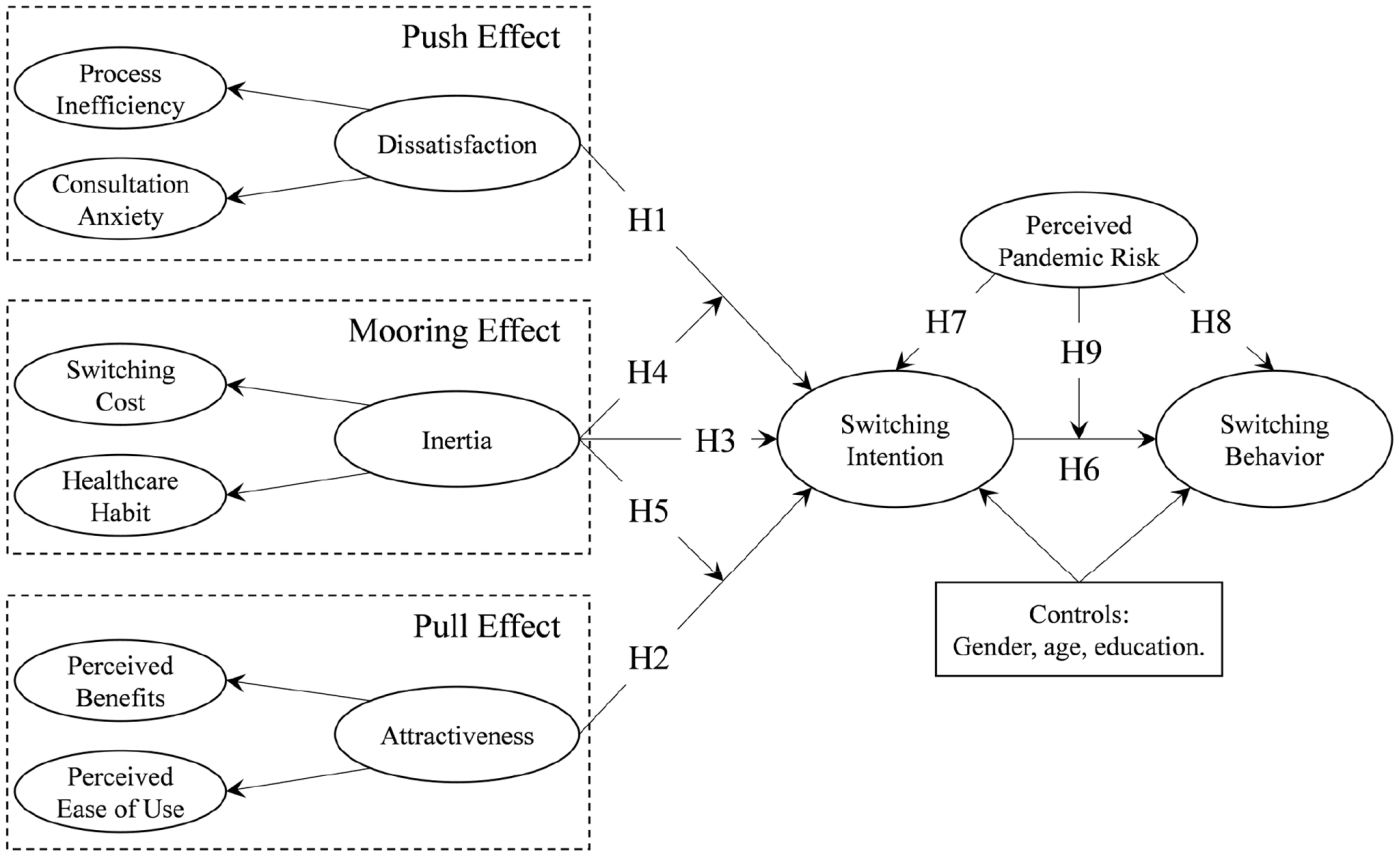
Research framework.

Considering that OHC is an alternative to offline healthcare services, the present study applies the PPM framework to conceptualize the switching of users from offline to online health consultation, thereby examining the push, pull, and mooring factors that drive such intention and behavior. The push, pull, and mooring factors are linked to the characteristics of offline healthcare services, OHC, and users, respectively. In addition, given the profound impact of the recent pandemic, the study also explores the effect of the perceived pandemic risk on the intention and behavior of the switching. Accordingly, a framework as shown in Figure 1 is developed.

### 3.1. Dissatisfaction with offline health consultation as a push effect

In a PPM framework, the push effect is normally the negative impact of the original service. For the switching from offline to online health consultation, the push effect corresponds to the inabilities or disadvantages of offline health services, which can be conceptualized as a latent construct of dissatisfaction. As has been confirmed by various PPM studies, dissatisfaction with the original service pushes users away (31, 33). In a study of OHC adoption, satisfaction with offline health services has been found to negatively influence the awareness of OHC which, in turn, further prevent the adoption (3).

Dissatisfaction with offline health services has been linked to a range of sources. Particularly, offline health services are often regarded as inefficient and time-consuming (37). For example, a survey in Iran revealed that waiting time accounts for more than 90% of the total time of outpatients’ visits (38). This is largely caused by the delayed starting time of physicians. Additionally, the consultation itself may lead to dissatisfaction. Otani et al. (39) found that courtesy and respect by nurses and physicians was pivotal in determining patient satisfaction. By investigating online reviews of patients, Shah et al. (40) also reported that the dissatisfaction of patients is heavily influenced by factors including doctors’ attitudes, communication, and unfriendly staff. As such, patients may feel anxious when consulting offline. Meanwhile, anxious patients are reported to feel less satisfied with offline consultation because they believe that physicians had provided not enough professional care and time (41). Accordingly, we consider the perceived process inefficiency and consultation anxiety of patients as the major reasons for their dissatisfaction with offline health service, and thus the following hypothesis is developed.

*H1*: Dissatisfaction with offline health services, including process inefficiency and consultation anxiety, has a positive effect on users’ intention to switch to OHC.

### 3.2. Attractiveness of online health consultation as a pull effect

The pull effect in a PPM framework is normally derived from positive factors of the alternative service, which are widely conceptualized as attractiveness. More specifically, the attractiveness of the alternative service is widely considered from two perspectives, namely the perceived benefits and perceived ease of use (30, 32, 33). In other words, if the alternative service could potentially provide more benefits and cost less effort to adopt, the users would be more likely to switch from the original service.

In the studies of OHC adoption, the perceived benefits and perceived ease of use are also widely explored. The perceived benefits normally refer to the utility of OHC, including usefulness for health management, time efficiency, and low medical expenses, especially in comparison to going to medical facilities (8, 10, 11). The ease of use describes how easily a system can be understood and accessed by users, and has been widely regarded as a key factor for the acceptance of IT systems (42, 43). While OHCs are hosted on online platforms, the ease of use of OHC systems has also been found to play a positive role in the adoption of OHCs (8, 9). Based on the literature, we consequently hypothesize that the perceived benefits and perceived ease of use lead to the attractiveness of OHC, which further pulls users to adopt OHC. In other words, we propose the following hypothesis.

*H2*: The attractiveness of OHC, including perceived benefits and perceived ease of use, has a positive effect on users’ intention to switch to OHC.

### 3.3. Behavioral inertia as a mooring effect

The mooring effect in a PPM framework captures the resistance to switching due to various reasons. This is in line with the status quo bias theory, that individuals’ decision-making is disproportionately biased toward maintaining their current status (44). The status quo bias is normally manifested by the concept of inertia, which refers to the “attachment to, and persistence of, existing behavioral patterns, even if there are better alternatives or incentives to change” [(45), p22]. For the scenarios involving users or consumers, the inertia is most often operationalized as switching cost and habit, such as in the switching between social networking sites (46), or between mobile applications (35). On the one hand, a high switching cost, including transition cost and sunk cost (previous commitment to the current behavior), would generally inhibit switching. On the other hand, the habit developed from previous behavior also prevents the switching since switching very often means adopting a new behavioral habit.

In considering switching between healthcare options, despite the possible awareness of the alternative (OHC), users may still decide to attend offline health consultation considering the switching cost (the time and effort devoted to getting familiar with offline consultation, and the potential time and effort to be spent for transition) and habit (being used to offline consultation). In the literature, switching costs and healthcare habits have been found to have negative effects on the intention to adopt OHC (11, 12). Similarly, we have the following hypothesis for the impact of inertia on switching behavior.

*H3*: The inertia of users’ healthcare usage, including switching cost and healthcare habit, has a negative effect on users’ intention to switch to OHC.

In the widely adopted definition, inertia is the tendency of maintaining current behavior in spite of the “incentives to change” (45). In the PPM framework, such incentives can be translated to the push and pull effects. Inertia, or, the mooring factor, is consequently believed to interact with push and pull factors. Generally, the mooring factor is expected to negatively moderate push and pull effects on switching intention. Such moderation role of the mooring factor has been confirmed in various PPM studies [e.g. (32, 46)]. In a study of OHC adoption, Zhang et al. (3) explored a similar moderation effect that offline healthcare habit enhances the negative impact of offline healthcare satisfaction on OHC awareness. This also supports the potential moderation role of inertia, as manifested by switching cost and offline healthcare habit, on the push (dissatisfaction with offline consultation) and pull (attractiveness of online consultation) effect. Accordingly, the following hypotheses are developed.

*H4*: Inertia negatively moderates the impact of offline service dissatisfaction on switching intention.

*H5*: Inertia negatively moderates the impact of OHC attractiveness on switching intention.

### 3.4. From intention to behavior

Behavioral intention is believed to be closely linked with actual behaviors. The theory of reasoned action argues that for simple and volitional behaviors, the intention is the most proximal determinant (47). Even for complex behaviors, the theory of planned behavior also posits a strong transition from intention to behavior, as long as the individual has control over the performance of the particular behavior (48). Such theories have also been applied to understand a wide range of health-related behaviors (49). Among various health-related behaviors, attending OHC is rather a volitional, or at least controllable, action. As such, we believe the switching intention significantly leads to the switching behavior from offline to online health consultation, which can be formalized as the following hypothesis.

*H6*: Intention to switch to OHC has a positive effect on the actual switching behavior.

### 3.5. Impact of perceived pandemic risks

The perceived pandemic risk induces problems in mental well-being and perceived uncertainty (50), which, in turn, significantly influence individuals’ intention to travel (51, 52). This is probably due to the common belief in social distancing as a measure to reduce pandemic risk. People thus tend to avoid face-to-face interactions. Consequently, evidence has been found that people have turned to online venues for shopping (53) and education (54). Attending offline health consultations also requires travel and face-to-face interaction, which potentially increases health risks. Considering the effectiveness of online health in reducing such risks (21), health-related behaviors have been largely migrated to online systems since the outbreak of the pandemic. It is reported that most of the mental healthcare practitioners in the Netherlands adopted online health systems on daily basis during COVID-19, in contrast to the very low use rate before the outbreak of the pandemic (15). Regarding patients, the daily visits to OHC platforms also saw a dramatic increase during the pandemic, and such an increase continued even after the pandemic got under control (5). As a consequence, we hypothesize that the perceived pandemic risk has a significant impact on the users’ decision regarding switching from offline to online health consultation, including direct effects on the switching intention and behavior, and a moderation effect on the relationship between such intention and behavior.

*H7*: Perceived pandemic risk has a positive effect on users’ intention to switch to OHC.

*H8*: Perceived pandemic risk has a positive effect on users’ actual behavior of switching to OHC.

*H9*: Perceived pandemic risk positively moderates the relationship between the intention and behavior of switching to OHC.

## 4. Methodology

To test the proposed hypotheses and model, a questionnaire was developed with detailed constructs and scale items, as reported in Table 2. The development of items on switching behavior was informed by seminal studies on the acceptance of information systems and technologies, because the switching behavior is essentially a discontinuance of offline health consultation and adoption of the online system. Accordingly, the items for measurements in the present study are adapted from previously tested sources. We measure the process inefficiency of offline health consultation from two aspects of difficulties in getting services, namely prolonged time cost and complicated process, inspired by Thompson et al. (55). Consultation anxiety items are adopted from the anxiety construct of Venkatesh et al. (56). Extensively tested items for perceived benefits and perceived ease of use are taken from Davis (42) and Moore and Benbasat (57). Switching cost is measured by items of transition cost and sunk cost taken from Polites and Karahanna (45) and Bansal et al. (58). Items for healthcare habit are composed according to Polites and Karahanna (45). Perceived pandemic risk items are adopted from Neuburger and Egger (59). Finally, switching intention and switching behavior are adapted from Venkatesh et al. (56) and Bansal et al. (58), respectively. All the items, except the ones for switching behavior, were measured via a five-point Likert scale, where 1 represents strongly disagree, while 5 represents strongly agree.

**Table 2:** Item descriptions and loadings.

Construct/Items	Loading	CR	AVE
Process inefficiency (55)		0.90	0.70
Consultations in hospitals take too much time waiting.	0.87		
Going to hospitals for consultation is so complicated.	0.83		
It is difficult to understand the procedure of consultation in hospitals.	0.80		
Going to hospitals involves too much time traveling.	0.83		
Consultation anxiety (56)		0.92	0.74
I feel apprehensive about going to hospitals.	0.84		
It scares me to think that I could misunderstand doctors’ instructions due to the time pressure of offline consultation.	0.88		
I hesitate to have offline health consultations for fear of the bad attitude of physicians and nurses.	0.88		
The face-to-face health consultation is somewhat intimidating to me.	0.85		
Perceived benefits (42)		0.88	0.65
I would find OHC useful to consult physicians anytime, anywhere.	0.85		
Using OHC would enable me to complete the consultation more quickly.	0.77		
Using OHC would enhance the effectiveness of my consultation.	0.83		
Using OHC would make it easier to find a physician that is more matched to my condition.	0.77		
Perceived ease of use (42, 57)		0.88	0.72
I would find it easy to use OHC platforms to acquire health knowledge.	0.78		
I would find it easy to use OHC platforms to acquire information about physicians.	0.88		
Overall, I believe that the OHC platforms are easy to use.	0.88		
Switching Cost (45, 58)		0.91	0.76
Learning how to use OHC would take too much time.	0.89		
I have already invested a lot of time in getting used to having health consultations in hospitals.	0.82		
Overall, I would spend a lot and lose a lot if I switched to OHC.	0.90		
Healthcare habit (45)		0.83	0.62
Whenever I need to have health consultations, I choose offline hospitals without even being aware of making the choice.	0.78		
Selecting offline hospitals for health consultation does not involve much thinking.	0.86		
Choosing offline hospitals for health consultation has been something I do unconsciously in the past.	0.73		
Perceived pandemic risk (59)		0.91	0.84
Although the COVID-19 restrictions are eased, the current situation still worries me.	0.92		
I fear that the virus will be carried by other patients in hospitals.	0.91		
Switching intention (56)		0.90	0.81
I predict I will use OHC when needed next time.	0.92		
I intend to use OHC to replace offline hospitals when possible.	0.89		
Switching behavior (58)		0.90	0.80
Did you use online platforms to have health consultations in the past 6 months? [Never (1) to always (5)].	0.90		
Did you use OHC platforms to buy medication in the past 6 months? [Never (1) to always (5)].	0.89		

The questionnaire was designed to be distributed via a well-developed platform, Wenjuanxing,^1^ where anonymous users participate in surveys. All the measurement items are developed in English at first and translated to Chinese since the platform is based in China. Before officially distributing the questionnaire, a small-scale pilot survey was firstly carried out. According to the pilot survey, the questionnaire does not contain any confusing items, and it normally takes about 3 to 5 min to complete the questionnaire. The official distribution collected a total of 365 responses during April 2022. A quality check was then performed, which excluded 47 invalid responses (e.g., incomplete, completed within 2 min, or having the same answers for every question). The remaining 318 valid responses are kept for the subsequent analysis. The demographic information of the valid responses is reported in Table 3. The respondents consist of 155 (48.7) males and 163 (51.3%) females, and are mostly college or university graduates (53.1%) aged between 31 to 40 (43.7%).

**Table 3:** Demographic information.

	Item	Frequency	Percentage (%)
Gender	Male	155	48.74
	Female	163	51.26
Age (years)	<30	104	32.70
	31–40	139	43.71
	41–50	48	15.09
	>50	27	8.49
Education	Junior high school or blow	28	8.81
	Senior high school	87	27.36
	College/university	169	53.14
	Master’s degree or above	34	10.69

## 5. Results

To analyze the collected responses, the partial least squares-structural equation modeling (PLS-SEM) was adopted, considering the developed formative constructs and the necessity of testing both the measurement model and the structural model. The analysis was conducted mainly in the software of SmartPLS.

### 5.1. Measurement model

Following Fornell and Larcker (60), we apply factor loadings, composite reliability (CR) scores, and average variance extracted (AVE) values to test the convergent validity of the reflectively measured scales. We also adopt the commonly used thresholds, including 0.7 for factor loadings and CR, and 0.5 for AVE values [e.g. (11, 50)]. As reported in Table 2, all scale items have factor loadings ranging from 0.73 to 0.92, which exceed the threshold of 0.7. The CR scores range from 0.83 to 0.92, and thus are also larger than the threshold of 0.7. The AVE values of all constructs ranging from 0.62 to 0.84 are larger than the threshold of 0.5. Such results indicate that the measurement model has a good reliability. We further compare the square roots of AVE values with the variance shared among constructs to assess the discriminant validity. Table 4 reports the square roots of AVE values for each construct as presented on the diagonal, while the off-diagonal values are correlations between the two constructs. It is suggested that all the square roots of AVE values are greater than the corresponding off-diagonal correlations, indicating a satisfactory level of discriminant validity.

**Table 4:** Discriminant validity.

	PI	CA	PB	PE	SW	HH	PR	SI	SB
Process Inefficiency (PI)	0.83								
Consultation Anxiety (CA)	0.61	0.86							
Perceived Benefits (PB)	0.70	0.55	0.80						
Perceived Ease of use	0.67	0.60	0.62	0.85					
Switching Cost (SC)	−0.72	−0.55	−0.68	−0.58	0.87				
Healthcare Habit (HH)	−0.50	−0.48	−0.47	−0.47	0.52	0.79			
Perceived pandemic Risk (PR)	0.68	0.52	0.67	0.54	−0.69	−0.50	0.92		
Switching Intention (SI)	0.69	0.60	0.71	0.72	−0.64	−0.55	0.60	0.90	
Switching Behavior (SB)	0.57	0.54	0.62	0.62	−0.56	−0.54	0.53	0.81	0.90

### 5.2. Structural model

**Figure 2:**
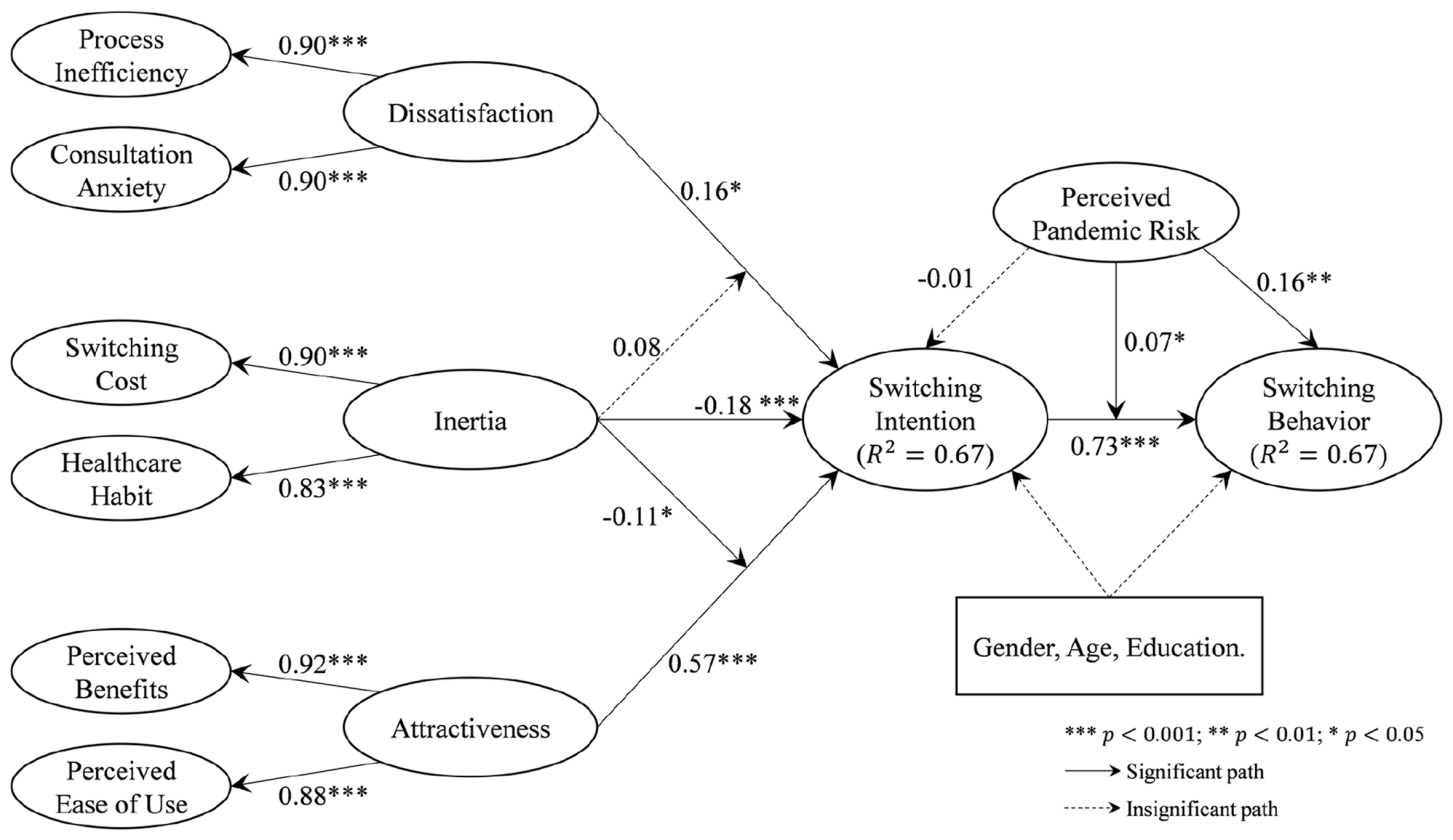
Results of the main effects model.

The results of the structural modeling are reported in Figure 2. In the proposed model, the push, pull, and mooring effects are conceptualized as second-order formative constructs, namely the dissatisfaction with offline service, the attractiveness of OHC, and the inertia of user behavior. As the results indicate, both dissatisfaction with offline service (β=0.16,p<0.05) and OHC attractiveness (β=0.57,p<0.001) show significant and positive impacts on the switching intention of users, indicating significant push and pull effects as expected. Thus, H1 and H2 are fully supported. Behavioral inertia shows a negative effect (β=−0.18,p<0.01) on switching intention, thereby supporting H3. While inertia is expected to play moderation roles for both push and pull effects, the negative moderation is found significant only for the pull effect (β=−0.11,p<0.05), that OHC attractiveness has a weaker effect on switching intention for users with strong behavioral inertia. Accordingly, H5 is supported while H4 is rejected.

As expected, the intention is closely linked to the actual behavior of switching (β=0.73,p<0.001), supporting H6. Such a link is also positively moderated by perceived pandemic risk (β=0.07,p<0.05), which means that the intention of switching to OHC is more likely to turn into actual behavior under the threats of pandemic risks. H9 is accordingly supported. Meanwhile, perceived pandemic risk also directly influences the switching behavior (β=0.16,p<0.01), thus supporting H8. However, the expected relationship between perceived pandemic risk on switching intention, that is, H7, is not supported.

Overall, the developed PPM framework explains 67% of the variance in switching intention, while switching intention and perceived pandemic risk can explain 67% of the variance in switching behavior.

**Figure 3:**
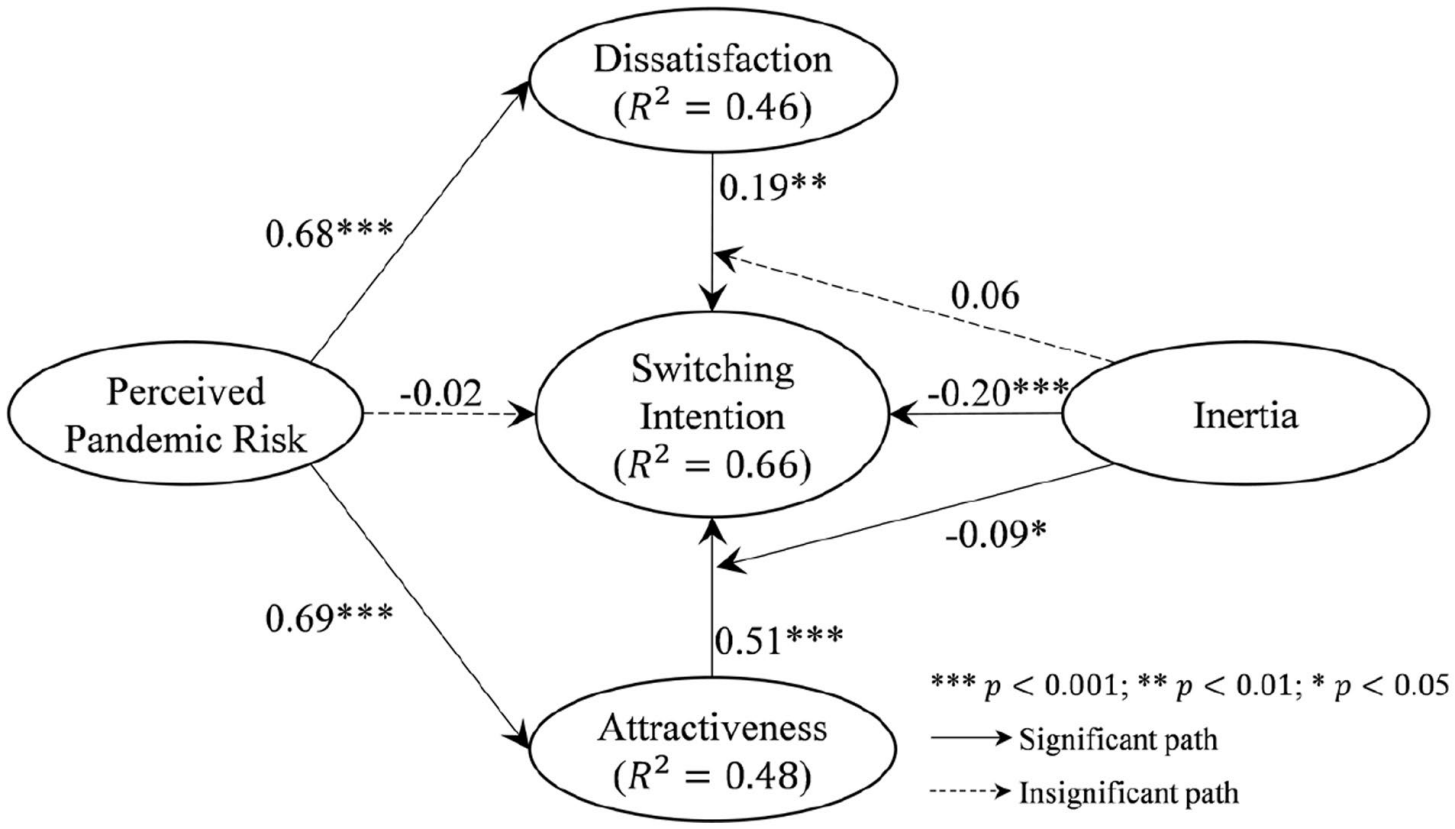
Results of the mediated effects of perceived pandemic risk on switching intention.

The non-significant relationship between perceived pandemic risk on switching intention was unexpected. However, a univariate linear regression of perceived pandemic risk on switching intention shows a significant positive impact (β=0.60,p<0.001). A possible reason for such insignificance in the full model could be the presence of push and pull factors. While perceived pandemic risk does not directly drive switching intention, it could contribute to perceived dissatisfaction toward offline services and the attractiveness of OHC, thereby indirectly promoting switching intention. Therefore, we further explore whether the impact of perceived pandemic risk on switching intention is mediated by the push and pull factors. Based on the original PPM model, we add two possible mediation paths via the push (dissatisfaction with offline service) and pull (attractiveness of online service) factors, respectively, resulting in a revised model as shown in Figure 3. The direct effect of perceived pandemic risk is still insignificant. However, perceived pandemic risk is reported to significantly influence users’ dissatisfaction with offline health consultation (β=0.68,p<0.001) and the attractiveness of OHC (β=0.69,p<0.001), which, in turn, leads to the switching intention. It is thus suggested that perceived pandemic risk has an indirect effect on users’ intention to switch from offline to online health consultation, as mediated by dissatisfaction with offline service and the attractiveness of online service.

## 6. Conclusions and discussions

### 6.1. Summary of findings

The objective of the present study is two-fold. On the one hand, the study focuses on exploring the role of perceived pandemic risk, during the current post-pandemic era, in individuals’ decisions to switching health consultation channels. On the other hand, we aim to integrate various influential factors for OHC adoption, including offline service factors, online service factors, and user-related factors, into a single PPM framework to analyze the switching decision from offline to online consultation.

The offline service factors, online service factors, and user-related factors are all influential for users’ switching intention. The inefficient consultation procedure and the anxiety potentially caused by the in-person consultation lead to dissatisfaction with offline health consultation, which produces push factors in favor of switching. The attractiveness of OHC mainly includes the perceived benefits (such as convenience and effectiveness) and perceived ease of use of the system, and pulls users to adopt OHC. Meanwhile, users with strong behavioral inertia, due to either the high transition and sunk costs, or their habit of going to medical facilities for consultation, tend not to switch to OHC. These users also receive weaker pull effects that the OHC attractiveness has less impact on their intention to switch to OHC.

While previous research demonstrated the significant impact of the pandemic on traveling (50–52), the present study further uncovers the pandemic’s influence on individuals’ health consultation behavior. Such an influence is three-fold. First, the perceived pandemic risk is found to have an indirect impact on the intention to switch from offline to online consultation, by increasing the users’ perception of offline service dissatisfaction and OHC attractiveness which, in turn, enhances the switching intention. Second, the perceived pandemic risk directly drives users’ actual behavior of switching to OHC. Third, the transition from switching intention to behavior is also enhanced by the perceived pandemic risk that users who intend to switch would be more likely to actually switch if they believe the pandemic risk to be high.

### 6.2. Theoretical contribution

The present study offers several contributions to the literature. First, the findings advance our understanding of health consultation behavior against the background of the COVID-19 pandemic. Several previous studies have focused on this subject. For example, Sutherland et al. (21) argue video consultations to be an effective approach during the COVID-19 pandemic to reduce the risks. Imlach et al. (22) explored patients’ satisfaction with OHC during the pandemic lockdown period. However, these studies regarded the pandemic as a study context, rather than developing a construct related to the pandemic to directly explore the pandemic’s influence on the users’ health consultation behavior. In contrast, the present study focuses on the perceived pandemic risk of users and thereby explores its influence on their intention and behavior of switching to OHC. Although as one of the most strictly regulated countries, China had eased its restrictions on domestic traveling during the survey period (i.e., April 2022). In such a post-pandemic era, the perceived pandemic risk is still found to be influential to drive users’ intention to switch to OHC. This is in line with conclusions from the literature that the pandemic risk leads to trailing impacts on travel (52). Attending offline health consultations also requires traveling, while OHC does not. High perceived pandemic risk thus leads to a stronger perception of the inconvenience of offline service, as well as stronger perceived benefits (travel avoidance) of online service. Accordingly, the perceived pandemic risk strongly associates (a mediated effect) with the intention of switching to OHC.

Second, the study provides theoretical explanations for the empirical observation of the increased OHC usage. Since the outbreak of the COVID-19 pandemic, many essential or inessential activities have moved to online venues, including various health-related activities (15). The dramatic increase in the usage of online health platforms and OHC is widely observed [49th Statistical Report on Internet Development in China (5, 18)]. This hinders an empirical link between the pandemic and OHC usage. However, the underlying mechanism for such increased OHC usage due to the pandemic is still lacking. This study depicts the influence of the pandemic via a construct of perceived pandemic risk, thereby establishing a theoretical linkage between the pandemic and OHC usage. It is revealed that the perceived pandemic risk not only directly encourages the actual adoption of OHC but also enhances the transition from intention to the behavior of using OHC. Accordingly, the results in the present paper could explain, from a theoretical perspective, the increase in OHC usage even during the post-pandemic era.

Last, we enrich the study of OHC adoption, by integrating and contextualizing the various influential factors into a PPM framework. In the literature on OHC adoption, factors related to OHC and users have been extensively investigated [e.g. (8–10)]. However, OHC platforms are developed to complement offline healthcare services. The factors related to offline service have thus also potential influence on the OHC adoption but have not been well explored. Zhang et al. (3) reported that satisfaction with offline healthcare services has a negative impact on the awareness of OHC. However, such satisfaction is measured via service satisfaction, system satisfaction, and information satisfaction, which are not contextualized for offline health consultations. Meanwhile, an integrated theoretical model is still lacking for the understanding of different roles of factors from different dimensions. The present study applies a PPM framework to conceptualize the influence of offline-related factors (push effect), OHC-related factors (pull effect), and user-related factors (mooring effect), thereby offering an integrated view of the drivers of OHC adoption. In particular, we contextualize the push effect (dissatisfaction with offline service) via the constructs of process inefficiency and consultation anxiety, which are identified as major inhibitors for offline consultation. The perceived pandemic risk and actual OHC behavior are also embedded into the framework, thereby providing a comprehensive understanding of the OHC intention and behavior in the post-pandemic era.

### 6.3. Practical implications

A number of practical implications are generated for traditional medical facilities, online health platforms, as well as relevant policymakers. Regarding traditional medical facilities, the present study suggests that process inefficiency and consultation anxiety push patients away. To prevent the loss of patients, traditional medical facilities need to focus on tuning the healthcare delivery procedures, thereby reducing the complexity and time cost for patients to access services. Meanwhile, a caring attitude and providing clear explanations and instructions on the condition can also largely improve patients’ satisfaction with traditional medical facilities. For online health platforms, the timely delivery of health services is a major attractiveness for patients. To embed toolkits to help with the matching between patients’ conditions and appropriate physicians is also an approach to enhance the perceived benefits of OHC. Additionally, a simple design of the system can ease the barriers to using OHC and thus attract more users.

From the viewpoint of policymakers, although OHC has its various benefits, it is not a replacement to traditional healthcare services, but rather a complement. Policymakers thus ought to find a balance for healthcare delivery via offline and online venues. To this end, a plausible approach is to enhance the pull effect (e.g., increase the usability and benefits of OHC), while weakening the push effect (e.g., regulate offline medical facilities regarding efficiency and courtesy). Meanwhile, in the current post-pandemic era, the perceived pandemic risk still acts as an inhibitor for general patients to attend offline health consultations. It is still necessary for policymakers to develop risk control mechanisms to enable patients’ choices between online and offline services according to their needs, instead of being concerned about pandemic risk.

### 6.4. Limitations and future research

The present study has several limitations that shall be further addressed in future research. First, we adopt a somewhat dichotomous approach to choice in healthcare. Patients can in fact choose to consult both in-person and online medical services. For example, patients can first consult a physician online and then decide whether to visit the physician according to their satisfaction with the online service (24), or patients use online follow-up services after their offline consultation (25). Accordingly, what factors influence patients’ decision to use such online-offline-combined health services shall be further explored. Second, previous literature has demonstrated the significant influence of disease type on patients’ decision of using OHC (25, 27). As such, the push, pull, and mooring factors could vary for patients with different diseases. For example, patients with high-privacy diseases may choose to use OHC because of privacy concerns, while patients with high-risk diseases may choose to go to offline medical facilities due to trust issues. A PPM model moderated by disease type needs to be developed and tested in future research. Last, the data were collected in online survey website which is based only in China. Such survey-based data collect could lead to inaccurate observation and biased conclusions. Explorations on actual behavior data from more general contexts are still needed to confirm and strengthen the findings of present study.

## Data availability statement

The raw data supporting the conclusions of this article will be made available by the authors, without undue reservation.

## Ethics statement

Ethical review and approval was not required for the study on human participants in accordance with the local legislation and institutional requirements. The ethics committee waived the requirement of written informed consent for participation.

## Author contributions

XP and LH designed the work and wrote the manuscript. XZ and LY collected and analyzed the data. All authors contributed to the article and approved the submitted version.

## Funding

This work was partially supported by the National Fund of Philosophy and Social Science of China (22CTQ017), the MOE project of Humanities and Social Sciences of China (21YJC870014), the Social Science Fund of Jiangsu Province (Grant no. 21TQC005), and the Natural Science Foundation of the Jiangsu Higher Education Institutions (Grant no. 21KJB120013).

## Conflict of interest

The authors declare that the research was conducted in the absence of any commercial or financial relationships that could be construed as a potential conflict of interest.

## Publisher’s note

All claims expressed in this article are solely those of the authors and do not necessarily represent those of their affiliated organizations, or those of the publisher, the editors and the reviewers. Any product that may be evaluated in this article, or claim that may be made by its manufacturer, is not guaranteed or endorsed by the publisher.

## Footnotes

^1^ https://www.wjx.cn/
